# Misspecification of confounder-exposure and confounder-outcome associations leads to bias in effect estimates

**DOI:** 10.1186/s12874-022-01817-0

**Published:** 2023-01-12

**Authors:** Noah A. Schuster, Judith J. M. Rijnhart, Lisa C. Bosman, Jos W. R. Twisk, Thomas Klausch, Martijn W. Heymans

**Affiliations:** 1 Department of Epidemiology and Data Science, Amsterdam Public Health Institute, Amsterdam UMC, Vrije Universiteit Amsterdam, De Boelelaan, 1117 Amsterdam, The Netherlands; 2 College of Public Health, University of South Florida, Tampa, FL USA

**Keywords:** Confounding, Confounder-adjustment, Linearity assumption, Confounder-exposure association, Confounder-outcome association, Multivariable regression analysis, Propensity score methods

## Abstract

**Background:**

Confounding is a common issue in epidemiological research. Commonly used confounder-adjustment methods include multivariable regression analysis and propensity score methods. Although it is common practice to assess the linearity assumption for the exposure-outcome effect, most researchers do not assess linearity of the relationship between the confounder and the exposure and between the confounder and the outcome before adjusting for the confounder in the analysis. Failing to take the true non-linear functional form of the confounder-exposure and confounder-outcome associations into account may result in an under- or overestimation of the true exposure effect. Therefore, this paper aims to demonstrate the importance of assessing the linearity assumption for confounder-exposure and confounder-outcome associations and the importance of correctly specifying these associations when the linearity assumption is violated.

**Methods:**

A Monte Carlo simulation study was used to assess and compare the performance of confounder-adjustment methods when the functional form of the confounder-exposure and confounder-outcome associations were misspecified (i.e., linearity was wrongly assumed) and correctly specified (i.e., linearity was rightly assumed) under multiple sample sizes. An empirical data example was used to illustrate that the misspecification of confounder-exposure and confounder-outcome associations leads to bias.

**Results:**

The simulation study illustrated that the exposure effect estimate will be biased when for propensity score (PS) methods the confounder-exposure association is misspecified. For methods in which the outcome is regressed on the confounder or the PS, the exposure effect estimate will be biased if the confounder-outcome association is misspecified. In the empirical data example, correct specification of the confounder-exposure and confounder-outcome associations resulted in smaller exposure effect estimates.

**Conclusion:**

When attempting to remove bias by adjusting for confounding, misspecification of the confounder-exposure and confounder-outcome associations might actually introduce bias. It is therefore important that researchers not only assess the linearity of the exposure-outcome effect, but also of the confounder-exposure or confounder-outcome associations depending on the confounder-adjustment method used.

**Supplementary Information:**

The online version contains supplementary material available at 10.1186/s12874-022-01817-0.

## Background

Unlike in randomized controlled trials, the observed exposure values in observational studies are often influenced by the characteristics of the study subjects. As a result, there might be an unintended difference in baseline characteristics between exposed and unexposed individuals. If these characteristics are also associated with the outcome, then these covariates are confounders of the exposure-outcome effect. In other words, a confounder is a common cause of the exposure and the outcome [1]. A simple comparison of the outcome between exposure groups then results in a biased effect estimate [2, 3]. Therefore, in observational studies, to obtain an unbiased estimate of the exposure effect it is necessary to remove the spurious part of the exposure-outcome effect caused by the confounders.

There are different methods to obtain confounder-adjusted exposure effect estimates, such as multivariable regression analysis and various propensity score (PS) methods. In multivariable regression analysis the confounders are added to the model in which the outcome is regressed on the exposure [4]. This way, the confounder-outcome association is controlled for when estimating the causal effect. In propensity score methods a balancing score is created which can subsequently be used to adjust, stratify, or weight the exposure-outcome effect [2, 5]. By creating this balancing score, the confounder-exposure association is removed and an unbiased exposure effect estimate can be obtained [6].

When multivariable regression analysis is used to adjust the relation between a continuous exposure and an outcome for a continuous confounder, both the exposure-outcome effect and the confounder-outcome association are assumed to be linear. It is common practice to assess the linearity assumption for the exposure-outcome effect and there is a substantial body of literature that covers this topic [4, 7]. However, various reviews found that the quality of the reporting of confounder adjustment methods is suboptimal [8–11] Often studies fail to explicitly report whether linearity was assessed [11]. When it is incorrectly assumed that the confounders are linearly related with the exposure and outcome (i.e., if the associations are misspecified), the exposure effect estimate might be over- or underestimated. Thus, in an attempt to remove bias, bias may actually be introduced. The bias that remains (or is introduced) after adjusting for confounding is also called *residual confounding* [7, 11, 12].

The aim of this paper is to demonstrate the importance of assessing the linearity assumption for confounder-exposure and confounder-outcome associations and the importance of correctly specifying these associations when the linearity assumption is violated. First, we describe how the linearity assumption can be assessed. Second, we provide an overview of methods that can be used to model non-linear effects. Third, we review four well-known confounder-adjustment methods and lay out their respective functional form assumptions. Fourth, we illustrate the importance of the correct specification of the confounder-exposure and confounder-outcome associations using a Monte Carlo simulation and an empirical data example. Fifth, we discuss methods that can be used to correctly specify the confounder-exposure and confounder-outcome associations.

### Examination of the linearity assumption

**Fig. 1:**
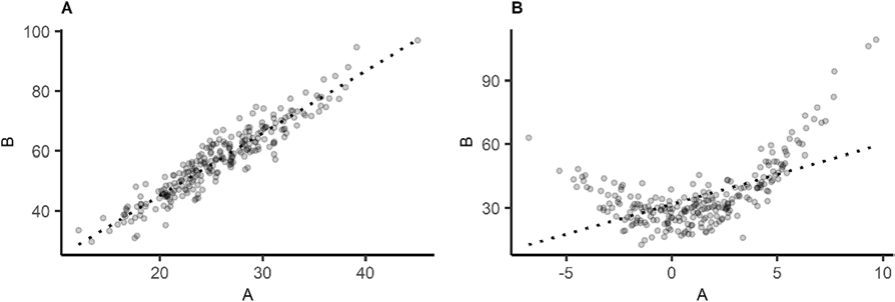
Hypothetical example of the relationship between continuous variables **A** and **B**, where each point represents an observation. **Panel** **a**: linear relationship. **Panel** **b**: non-linear relationship. The dotted line represents the linear regression line for the relationship between variables **A** and **B**

Suppose that variable A represents a continuous independent variable, variable B represents a continuous dependent variable. There are several methods to assess the linearity of the association between variables A and B. A first method is visual inspection: a scatterplot with variable A on the X-axis and variable B on the Y-axis provides an indication of the nature of the relationship between A and B [13]. Figure 1 provides a hypothetical example of a linear relationship between variables A and B (panel A), and a non-linear relationship between those variables (panel B). In both panels, the circles represent the observed data and the dotted line represents the linear regression line, i.e., the line that describes a linear relationship between variables A and B. In panel A, the regression line fits the data well, because the circles in the scatterplot resemble a straight line. In panel B, however, the linear regression line is not a good representation of the non-linear relationship between A and B, because the circles in the scatterplot do not resemble a straight line. Then, failing to model the A-B association as non-linear results in a biased estimate of this association.

A second method to assess linearity is to categorize the continuous variable A into multiple groups of equal sizes, e.g., based on tertiles or quartiles of the distribution of variable A. Subsequently, variable B is modelled as a function of a categorized variable A. If the regression coefficients corresponding to the categories of variable A do not increase linearly, then this indicates that the A-B association is non-linear [7]. A third method to assess linearity is the addition of a non-linear term for variable A, e.g., a quadratic term, to the model. When adding a non-linear term to the model, variable B is modelled as a function of variable A and the non-linear term of variable A. If the A-B association is truly linear, then the coefficient corresponding to the non-linear term will be zero [4]. Often, statistical significance of the non-linear term is used as a threshold to determine whether the linearity assumption is violated.

Although in this paper we focus on linear regression models, the linearity assumption is also applicable to continuous independent variables in generalized linear models, such as logistic regression models. In generalized linear models the linearity assumption can be checked using the two non-visual methods, i.e., categorization of the independent variable and by adding non-linear terms for the independent variable.

### Modelling non-linear associations

There are several methods that can be used to model the non-linear associations, such as categorization, the use of non-linear terms and the use of spline functions. An overview of commonly used methods for modelling non-linear associations, their application and advantages and disadvantages can be found in Table 1.

**Table 1:** Methods to approximate true non-linear effects

Method	Explanation	Advantages	Disadvantages
Categorization	The confounder is grouped (e.g. on pre-specified percentile values such as quartiles) and subsequently the outcome is regressed on the exposure and the now categorical confounding variable	Easy to apply	Homogeneity of the effects is assumed within groups, resulting in severe loss of information and possibly residual confounding
Non-linear terms	The outcome is regressed on the confounder and the non-linear term of that same confounder, e.g., a quadratic term	Easy to apply Adding non-linear order terms increases the flexibility of the model	Coefficients are difficult to interpret^*^
Linear spline regression	First, the confounding variable is categorized and subsequently a first power function is fitted for each category separately. After fitting the spline functions these are added to the regression model	Good approximation of the true effect Coefficients are easy to interpret	
Restricted cubic spline regression	Same as linear spline regression, but instead a more flexible third power function is fitted for each category separately. To avoid instability in the tails where there’s not much data, *restricted* cubic splines are often used where at the tails a line is fitted rather than a curve.	Good approximation of the true effect Adding splines increases the flexibility of the model	Coefficients are difficult to interpret^*^

^*^ This is not a hindrance when these methods are used to model non-linear confounder-exposure or confounder-outcome associations as the corresponding coefficients will not be interpreted

A first method that is sometimes used to model non-linear associations is categorization [14–17]. Suppose that the confounder-outcome association is non-linear. With categorization the subjects in the dataset are being categorized based on their values on the continuous confounder variable. Groups can be created based on substantively meaningful cut-off points or statistical cut-off points (e.g., tertiles or quartiles). Subsequently, *n*-1 dummy variables are created based on the categorical confounder variable, where *n* represents the number of categories. For example, if the variable consists of four categories, then three dummy variables are created. The reference group is coded as 0 in each of these dummy variables, while in each dummy variable one of the other groups is coded as 1. Subsequently, the outcome is regressed on the dummy variables, where the regression coefficient for each dummy variable represents the difference in the outcome between the group coded as 1 and the reference group. A disadvantage of modelling a non-linear association by categorization is that the magnitude of the association is assumed to be the same for all subjects a within a specific group [14–17]. Therefore, a potential non-linear association *within* a category is not captured in the analysis.

Another method that can be used to model the non-linear associations is the inclusion of non-linear terms, such as quadratic or cubic terms, in the regression model [18]. Suppose that the confounder-outcome association follows a quadratic shape, then this can be modelled by regressing the outcome on the original confounder variable and a quadratic term for the confounder. Adding non-linear terms increases the flexibility of the model, but also reduces the interpretability of the results [18]. However, using non-linear terms to approximate the non-linearity of the confounder-exposure or confounder-outcome association does not affect the interpretability of the exposure effect.

Spline regression is another method that can be used to model non-linear associations. Two types of splines that are commonly used are linear splines and restricted cubic splines. With spline regression, the confounding variable is also categorized, but instead of assuming that the association is of the same magnitude for all subjects in a specific category, a regression line is estimated for each category [4, 13, 19]. Depending on whether linear or restricted cubic splines are used, the estimated regression line is linear or non-linear, respectively. The cut-off points in between categories are called *knots*. A 1-knot spline function is based on two categories, a 2-knot spline function on three categories, a 3-knot spline function on four categories, etcetera. Detailed information on the estimation of splines can be found elsewhere [20]. Like with non-linear terms, the interpretation of the coefficients can be complicated when spline functions are used [13]. However, because we are not necessarily interpreting the coefficients of the confounder-exposure or confounder-outcome associations, spline functions are a good and efficient way to approximate the non-linear shapes of those associations.

### Confounder-adjustment methods

Studies are often interested in estimating the average effect of an exposure on an outcome. In terms of potential outcomes, the average effect of the exposure on the outcome is defined as the difference between two expected potential outcome values under two exposure values, i.e., *E*[*Y*(1) − *Y*(0)]. To obtain an unbiased estimate of this exposure effect it is necessary to adjust for any confounding. In this study we discuss four confounder-adjustment methods: multivariable regression analysis, covariate adjustment using the propensity score (PS), inverse probability weighting (IPW) and double robust (DR) estimation. As assessing the linearity assumption for the exposure-outcome effect is common practice, throughout this paper we assume that the exposure-outcome effect is always correctly specified as linear. However, we believe that the information in this paper also applies to models in which the exposure-outcome effect is (correctly specified as) non-linear. Table 2 shows which association (i.e., the confounder-exposure or the confounder-outcome association, or both) has to be correctly specified for each method in order to obtain unbiased exposure effect estimates.

**Table 2:** Confounder-adjustment methods and the association(s) that need to be correctly specified to obtain an unbiased estimate of the exposure effect

Confounder-adjustment methods	Confounder-exposure association	Confounder-outcome association
Multivariable regression analysis	n/a	√
Covariate adjustment using the PS^§^	√	√^*^
IPW^§^	√	n/a
DR estimation^§^	Both associations need to be specified but estimators are consistent if either is *correctly* specified	

Abbreviations: *PS* propensity score, *IPW* inverse probability weighting, *DR* double robust, *PS*-outcome effect, n/a: not applicable, requires a correctly specified propensity score (i.e., the log odds of the exposure is linear in the confounders)

### Multivariable regression analysis

With multivariable regression analysis, the outcome is modelled as a function of the exposure and the confounders [4] (eq. 1):

1 $$E\left(Y|X,C\right)={i}_1+{\beta}_1X+{\beta}_2{C}_1+\dots +{\beta}_{n+1}{C}_n$$

where *Y* and *X* represent the continuous outcome and a dichotomous exposure, respectively, and *i*_1_ represents the intercept term. *β*_1_ is the multivariable confounder-adjusted exposure effect estimate and *β*_2_ to *β*_*n* + 1_ are the coefficients that correspond to the continuous confounding variables *C*_1_ to *C*_*n*_.

Multivariate regression analysis adjusts for confounding of the exposure-outcome effect by adding confounders *C*_1_ to *C*_*n*_ to the equation [4, 13]. If there are no unobserved confounders and the linear regression model in eq. 1 is correctly specified, then parameter *β*_1_ is equal to the average treatment effect E [Y(1)-Y(0)] [21].

In eq. 1, a linear association is assumed between the exposure and the outcome, and between each confounding variable and the outcome [13]. The confounder-exposure association is not modelled, therefore no assumptions are made about the functional form of that association.

#### Propensity score adjustment

The PS is the predicted probability of endorsing exposure (eq. 2):

2 $$PS=P\left(X=1|{C}_1,\dots, {C}_n\right)=\frac{1}{1+{e}^{-\left({i}_2+{\lambda}_1{C}_1+\dots +{\lambda}_n{C}_n\right)}}$$

where *X* represents the dichotomous exposure, *i*_2_ is the model intercept and *λ*_1_ to *λ*_*n*_ are regression coefficients corresponding to confounders *C*_1_ to *C*_*n*_.

The propensity score is estimated in two steps. First, the exposure is modelled as a function of the confounders *C*_1_ to *C*_*n*_ using a logistic regression model. Second, each individual’s predicted probability of endorsing the exposure is estimated, which is the propensity score [2, 6, 22].

The PS can be used in different ways to adjust for confounding. In this paper we discuss three of these methods: covariate adjustment with the PS, inverse probability weighting and double robust estimation. All three methods assume that the propensity score is correctly specified, i.e., that the log odds of the exposure is linear in the confounders. Details on the computation of the PS in general and other PS methods such as matching and stratification can be found elsewhere [2, 6, 22–29].

#### Covariate adjustment using the propensity score

Because the PS contains information on the confounders, it is possible to adjust for confounding by modelling the outcome as a function of the exposure and the PS [2, 22]. Thus, instead of conditioning on confounding variables *C*_1_ to *C*_*n*_ as in eq. 1, we now condition on the PS (eq. 3):

3 $$E\left(Y|X, PS\right)={i}_3+{\beta}_1^{\ast }X+{\beta}_2^{\ast } PS$$

where *Y* and *X* represent the continuous outcome and the dichotomous exposure, respectively, and *i*_3_ represents the intercept term. ${\beta}_1^{\ast }$ is the PS confounder-adjusted exposure effect estimate and ${\beta}_2^{\ast }$ is the coefficient that corresponds to the propensity score *PS*.

Because in eq. 3 the outcome is regressed on the exposure and the propensity score, linearity assumptions apply both to the exposure-outcome effect and the PS-outcome association. Whereas all PS methods require the PS to be adequately specified, this is the only PS method that additionally makes assumptions about the linearity of the PS-outcome association [2, 4].

#### Inverse probability weighting

Inverse probability weighting uses weights based on the PS to create a pseudo-population in which each confounder combination is balanced between the exposed and unexposed groups. When there is perfect confounder balance between the groups there is no longer an association between confounders *C*_1_ to *C*_*n*_ and the exposure [4]. With weighting, individuals who are underrepresented get larger weights assigned, whereas individuals who are overrepresented get smaller weights assigned.

For exposed individuals the weight is calculated as $\frac{1}{PS}$, whereas for unexposed individuals the weight is calculated as $\frac{1}{1- PS}$ [2, 30]. A potential issue with IPW is that the weights can be unstable. This is because individuals with a PS close to 0 receive very large weights, whereas individuals with a PS close to 1 receive very small weights. Subjects with these large weights will then dominate the weighted analysis, resulting in a large variance of the IPW estimator [31]. As an alternative, stabilized weights have been proposed [2]. This reduces the weights of the treated individuals with a small PS and the untreated individuals with a large PS. For exposed individuals, these stabilized weights are calculated as $\frac{p}{PS}$ and for unexposed individuals stabilized weights are calculated as $\frac{1-p}{1- PS}$, with *p* being the probability of exposure without considering the confounders [2]. After calculating the weights for all individuals the IPW confounder-adjusted exposure effect is estimated by performing a weighted regression analysis with the exposure as the only independent variable.

IPW does not make any linearity assumptions about the confounder-outcome or PS-outcome association [29]. Thus, IPW only assumes a correctly specified propensity model. If the propensity model is misspecified this results in inappropriate weights and possibly a biased IPW confounder-adjusted exposure effect estimate [32].

#### Double robust estimation

Double robust estimation combines multivariable regression analysis and IPW and is done in two steps: first, a propensity model is specified and stabilized weights are calculated. Second, a weighted analysis is conducted in which the outcome is regressed on the exposure *and* the confounders.

Because the model is weighted by the stabilized weights, an adequately specified propensity model is needed. In addition, because the confounders are included in the regression analysis, linearity assumptions about the confounder-outcome association are made. However, only one of these two associations (i.e., either the confounder-exposure associations in the propensity model or the confounder-outcome associations in the multivariable regression model) has to be correctly specified to obtain an unbiased exposure effect estimate [29, 32, 33]. However, if both effects are misspecified, the DR exposure effect estimate may be even more biased than the estimate of a less robust single confounder-adjustment method such as multivariable regression or IPW [34, 35].

### Simulation study

#### Simulation methods

A simulation study was designed to assess and compare the performance of the four confounder-adjustment methods. Four different scenarios were considered based on the (mis)specification of the confounder-exposure and confounder-outcome association (see Table 3). The R programming language version 4.0.3 was used to generate and analyse the data [36].

**Table 3:** Overview of simulated scenarios

Scenario	Confounder-exposure association	Confounder-outcome association
Scenario 1	Correctly specified	Correctly specified
Scenario 2	Correctly specified	Misspecified
Scenario 3	Misspecified	Correctly specified
Scenario 4	Misspecified	Misspecified

To model both misspecified and correctly specified confounder-exposure and confounder-outcome associations, first two continuous confounders were generated. Confounder *Z* was generated from a standard normal distribution, and confounder *C* was its corresponding squared term. The dichotomous exposure was generated from a binomial distribution conditional on confounder *Z* and its squared term *C* (eq. 4), and the continuous outcome was a function of the exposure and confounders *Z* and *C* (eq. 5).

4 $$P\left(X=1|Z,C\right)=\frac{1}{1+{e}^{-\left({i}_4+{\theta}_1Z+{\theta}_2C\right)}}$$

5 $$E\left(Y|X,Z,C\right)={i}_5+{\gamma}_1X+{\gamma}_2Z+{\gamma}_3C$$

This way, the exposure and the outcome had a quadratic relation with each of the confounders. Next, we estimated the confounder-adjusted exposure-outcome effect using the four confounder-adjustment methods. In the scenarios in which the non-linearity of the confounder-exposure and confounder-outcome association were correctly specified, the analysis was adjusted for confounders *Z* and *C*. This way, the underlying quadratic relation was modelled. In the scenarios in which the effects were misspecified, only confounder *Z* was included in the analysis. This way, only the incorrect linear relation was modelled. Sample sizes were 200, 500 and 1000. The parameter value for the exposure-outcome effect was set to 0.59 to mimic a large effect size. The parameter values for the confounder-exposure and confounder-outcome association were set to −0.14, −0.39, − 0.59 and 0.14, 0.39 and 0.59 to mimic negative and positive small, medium and large effect sizes, respectively [37]. In total, 72 conditions were simulated (4 scenarios; 3 sample sizes; 6 confounder-exposure and confounder-outcome effect sizes) with 1000 repetitions per condition, resulting in 72,000 observations.

The performance of the confounder-adjustment methods was compared based on the absolute bias (AB) and the relative bias (RB) [38]. AB is the absolute difference between the estimated exposure effect and the true exposure-outcome effect of 0.59. RB is the ratio of AB to the true exposure-outcome effect [38, 39]. For both performance measures a lower score corresponds to a better performance. The simulation code is available in additional file 1.

In additional file 2 we show an extra condition in which the direction of the exposure effect changes if the non-linearity of the confounder-exposure and confounder-outcome associations is not modelled correctly.

#### Simulation results

Table 4 shows the mean estimated exposure effect, AB and RB for all models across the four simulated scenarios based on a sample size of 500 and positive confounder-exposure and confounder-outcome associations. Results for sample sizes 200 and 1000 can be found in additional files 3 and 4, respectively.

**Table 4:** Model performance across all simulated scenarios, *n* = 500

	Parameter values for the confounder-exposure and confounder-outcome associations								
	0.14			0.39			0.59		
	$\hat{\beta}$	AB	RB	$\hat{\beta}$	AB	RB	$\hat{\beta}$	AB	RB
**Scenario 1: correct specification of cx-association & correct specification of cy-association**									
Multivariable regression analysis	0.5900	0.0000	0.0000	0.5900	0.0000	0.0000	0.5900	0.0000	0.0000
Covariate adjustment using the PS	0.5901	0.0001	0.0001	0.5907	0.0007	0.0012	0.5909	0.0009	0.0015
Stabilized IPW	0.5903	0.0003	0.0005	0.6030	0.0130	0.0220	0.6417	0.0517	0.0876
DR estimation	0.5900	0.0000	0.0000	0.5900	0.0000	0.0000	0.5900	0.0000	0.0000
**Scenario 2: correct specification of cx-association & misspecification of cy-association**									
Multivariable regression analysis	0.6263	0.0363	0.0615	0.8051	0.2151	0.3646	0.9859	0.3959	0.6711
Covariate adjustment using the PS	0.5901	0.0001	0.0001	0.5907	0.0007	0.0012	0.5909	0.0009	0.0015
Stabilized IPW	0.5903	0.0003	0.0005	0.6030	0.0130	0.0220	0.6417	0.0517	0.0876
DR estimation	0.5905	0.0005	0.0008	0.6064	0.0164	0.0278	0.6465	0.0565	0.0957
**Scenario 3: misspecification of cx-association & correct specification of cy-association**									
Multivariable regression analysis	0.5900	0.0000	0.0000	0.5900	0.0000	0.0000	0.5900	0.0000	0.0000
Covariate adjustment using the PS	0.6267	0.0367	0.0622	0.8147	0.2247	0.3808	1.0155	0.4255	0.7212
Stabilized IPW	0.6276	0.0376	0.0638	0.8339	0.2439	0.4134	1.0676	0.4776	0.8096
DR estimation	0.5900	0.0000	0.0000	0.5900	0.0000	0.0000	0.5900	0.0000	0.0000
**Scenario 4: misspecification of cx-association & misspecification of cy-association**									
Multivariable regression analysis	0.6263	0.0363	0.0615	0.8051	0.2151	0.3646	0.9859	0.3959	0.6711
Covariate adjustment using the PS	0.6267	0.0367	0.062	0.8147	0.2247	0.3808	1.0155	0.4255	0.7212
Stabilized IPW	0.6276	0.0376	0.0638	0.8339	0.2439	0.4134	1.0676	0.4776	0.8096
DR estimation	0.6273	0.0373	0.0533	0.8261	0.2361	0.4002	1.0456	0.4556	0.7723

Abbreviations: *n:* sample size, *cx*-association: confounder-exposure association, *cy*-association: confounder-outcome association, $\hat{\beta}$: mean estimated exposure effect, *AB:* absolute bias, *RB:* relative bias, *PS:* propensity score, *IPW:* inverse probability weighting, *DR:* double robust

In scenario 1, where both the confounder-exposure and confounder-outcome associations were correctly specified, multivariable regression analysis, PS adjustment and DR estimation all performed well. When the confounder-outcome association was misspecified (scenario 2), multivariable regression analysis and DR estimation resulted in biased exposure effect estimates. PS adjustment still performed well, but had the PS-outcome association been misspecified as well, then residual bias would also have been observed for that method. In both scenarios 1 and 2, bias was observed for IPW as IPW is a large sample technique [3]. Increasing the sample size resulted in exposure effect estimates closer to the true effect. In scenario 3, where the confounder-exposure association was misspecified but the confounder-outcome association was correctly specified, multivariable regression analysis and DR estimation performed well, whereas PS adjustment and IPW resulted in biased exposure effect estimates. When both associations were misspecified (scenario 4), all methods resulted in biased exposure effect estimates. In all scenarios, the amount of bias depended on the strength of the confounder-exposure and confounder-outcome associations: the weaker the associations were, the less biased was observed. The same patterns can be observed for negative confounder-exposure and confounder-outcome associations. For detailed results see additional file 5.

### Empirical data example

To demonstrate the consequences of misspecification of the confounder-exposure and confounder-outcome association we used an illustrative example from the Amsterdam Growth and Health Longitudinal Study (AGHLS). The AGHLS is an ongoing cohort study that started in 1976 to examine growth and health among teenagers. In later measurement rounds, health and lifestyle measures, determinants of chronic diseases and parameters for the investigation of deterioration in health with age were measured [40]. For this demonstration we use data collected in 2000, when the participants were in their late 30s.

Using data from the AGHLS, we estimated the effect of overweight (BMI ≥ 25) on systolic blood pressure. We adjusted this effect for confounding by alcohol consumption (measured in number of glasses per week) and cardiorespiratory fitness (VO2max). Only subjects with complete data on all variables were included in the analyses (*n* = 359). Note that this data example is included for illustrative purposes only and therefore represents a simplified scenario. In reality, it is likely that there will be additional confounders and time-varying confounders. As a result, substantive interpretations should be approached with caution.

First, we examined the linearity of the confounder-exposure and the confounder-outcome associations. We did this by categorizing alcohol consumption and cardiorespiratory fitness, and separately regressing overweight and systolic blood pressure on the categorized confounders. In both cases, the regression coefficients corresponding to the categories of alcohol consumption and respiratory fitness did not increase linearly. Thus, both confounder-exposure and confounder-outcome associations were non-linear. There were no violations of the linearity assumption for the exposure-outcome effect, as systolic blood pressure was compared across only two groups (i.e., healthy weight and overweight). Second, to demonstrate the consequences of misspecification, we modelled systolic blood pressure as a function of overweight, adjusting for alcohol consumption and cardiorespiratory fitness. We did this first by (falsely) assuming a linear relation between the confounders and overweight and between the confounders and systolic blood pressure. Next, we took these non-linear associations into account by adjusting for alcohol consumption and cardiorespiratory fitness using 3-knot restricted cubic spline (RCS) regression, which has the ability to.

fit non-linear shapes. A detailed explanation of RCS regression can be found elsewhere [4]. Although implementing RCS regression might still not equal perfect specification of both effects, it provides a better representation of the true non-linear relations than simply assuming linear confounder-exposure and confounder-outcome associations.

The results of the analyses can be found in Table 5. Across all four methods, the exposure effects were smaller in magnitude when the confounder-exposure and confounder-outcome associations were modelled as non-linear. Given that in our example the confounder-exposure and confounder-outcome associations were non-linear, the exposure effects were overestimated when the confounder-exposure and confounder-outcome associations were incorrectly modelled as linear.

**Table 5:** The effect of overweight on systolic blood pressure, adjusted for alcohol consumption. 2nd column: linear models in which the confounder-exposure and/or confounder-outcome associations are modelled as linear. 3rd column: spline models in which the confounder-exposure and/or confounder-outcome associations are modelled as non-linear

	Linear models	Non-linear spline models
	β (95% CI)	β (95% CI)
Multivariable regression analysis	3.589 (0.686; 6.493)	3.022 (0.136; 5.908)
Covariate adjustment using the PS	3.739 (0.822; 6.656)	3.062 (0.164; 5.960)
Stabilized IPW	4.121 (1.110; 7.132)	3.813 (0.807; 6.819)
DR estimation	3.983 (1.262; 6.704)	3.585 (0.879; 6.291)

Abbreviations: *PS:* propensity score, *IPW:* inverse probability weighting, *DR:* double robust, *β:* regression coefficient, *CI:* confidence interval

## Discussion

This paper aimed to demonstrate the importance of assessing the linearity assumption for confounder-exposure and confounder-outcome associations and the importance of correctly specifying these associations when the linearity assumption is violated. If these associations are incorrectly specified as linear, then bias might be introduced in an attempt to remove bias. Our simulation study showed that bias is introduced if the confounder-exposure and/or the confounder-outcome association are misspecified. The amount of bias also depended on the confounder-adjustment method and the strength of the confounder-exposure and confounder-outcome association. We also illustrated how misspecification of the confounder-exposure and/or confounder-outcome associations biases exposure-outcome effect estimates our empirical data example. The simulation study and the empirical data example both showed that merely adjusting for confounding is not enough, but that correct specification of *all* effects in the model is crucial to obtain unbiased exposure effect estimates.

### Reporting of confounding

The results in this paper demonstrate that misspecification of the confounder-exposure and confounder-outcome associations may lead to additional bias. However, in practice residual confounding may often go unnoticed, as inappropriate reporting makes it difficult to assess the reliability and validity of study results. In 2007 the STROBE (Strengthening the Reporting of Observational Studies in Epidemiology) initiative published a checklist of items that should be addressed in reports of observational studies, including two items that address confounding (9 ‘Bias’ and 12 ‘Statistical methods’) [41]. The explanatory and elaboration document of STROBE acknowledges that adjusting for confounding may involve additional assumptions about the functional form of the studied associations [42]. Despite the publication of the STROBE checklist, the overall quality of reporting of confounding remains suboptimal [9, 43]. To increase transparency on the risk of residual confounding, we advise researchers to report how the functional form of the confounder-exposure and confounder-outcome association was assessed and taken into account.

### Limitations

The simulation study in this paper is a simplified representation of real world scenarios. We adjusted for one confounder, whereas in reality there might be multiple confounders. If there are multiple confounders, then the confounder-exposure and confounder-outcome association of each of the confounders needs to be assessed and non-linear effects need to be modelled for confounders that are not linearly related to either the exposure or the outcome. In the PS methods, the PS-outcome association was linear, so no additional bias was observed in scenarios in which the confounder-outcome association was misspecified. However, if the PS-outcome association is also misspecified, residual bias would be observed. Therefore, the linearity of the relation between the PS and the outcome should always be checked. IPW is known to perform less well in small samples, which was also confirmed in our simulation [3]. Last, in this paper we assume associations are either misspecified or correctly specified, whereas in reality, naturally, everything exists in shades of grey. In addition, there are other important contributors to bias in the exposure effect estimate that researchers should be aware of, such as omitted confounders, adjustment for colliders, and measurement error in the confounders. A limited theoretical understanding of factors that influence exposures and outcomes may cause researchers to overlook important confounders or to adjust for a collider (i.e., a variable that is influenced by both the exposure and outcome). In both situations the estimate of the exposure effect will be biased [44]. Finally, there may be residual confounding when the confounders are measured with error [45].

## Conclusion

To summarize, in this study we showed the importance of correctly specifying the confounder-exposure and confounder-outcome associations to obtain unbiased exposure effect estimates. When these effects are misspecified, bias might actually be introduced in an attempt to remove bias. Thus, to estimate unbiased effects it is important to examine the linearity of the confounder-exposure or confounder-outcome association depending on the confounder-adjustment method used and to adjust the model accordingly.

## Supplementary Information

**Additional file 1.**

**Additional file 2.**

**Additional file 3.**

**Additional file 4.**

**Additional file 5.**

## Data Availability

1. The dataset analyzed in the empirical data example is available from the corresponding author on reasonable request. Human data from the AGHLS can be requested through the website https://aghls.wordpress.com/collaborate/. The code for the simulated dataset is included in this study in additional file 1

## Abbreviations

AB: absolute bias
AGHLS: Amsterdam Growth and Health Longitudinal Study
CI: confidence interval
DR: double robust
IPW: inverse probability weighting
n/a: not applicable
PS: propensity score
RB: relative bias
RCS: restricted cubic spline
STROBE: Strengthening the Reporting of Observational Studies in Epidemiology
