# Supplementary material for: Misspecification of confounder-exposure and confounder-outcome associations leads to bias in effect estimates
Source: BMC Med Res Methodol. 2023 Jan 12;23:11. doi: 10.1186/s12874-022-01817-0 (PMC9835340; doi:10.1186/s12874-022-01817-0)
Supplement: Supplementary file 1 — Additional file 1. [file 12874_2022_1817_MOESM1_ESM.docx]

**Additional file A Simulation code**

*Step 1 – Generate data*

generate_data <- function(seed, reps, n, ix, iy, cx, xy, cy){

# define total number of rows required to store data

rows <- reps * n

# create data frame to store data in

df <- as.data.frame(matrix(NA, nrow = rows, ncol = 13))

colnames(df) <- c("ID", # ID through entire data set

"repnr", # for each repetition

"ID_repnr", # ID through each repetition

"n", # number of observations

"ix", # intercept exposure

"iy", # intercept outcome

"cx", # confounder-exposure effect

"xy", # exposure-outcome effect

"cy", # confounder-outcome effect

"C", # continuous confounder (correctly specified)

"Z", # continuous confounder (misspecified)

"X", # dichotomous exposure

"Y") # continuous outcome

# define simulation parameters

df[, "ID"] <- seq(1:rows)

df[, "repnr"] <- rep(1:reps, each = n)

df[, "ID_repnr"] <- rep(seq(1, n), reps)

df[, "n"] <- n

# define intercepts ix and iy

df[, "ix"] <- ix

df[, "iy"] <- iy

# define coefficients cx, xy and cy

df[, "cx"] <- cx

df[, "xy"] <- xy

df[, "cy"] <- cy

# generate confounder Z from a standard normal distribution

# with mean = 0 and sd = 1 (misspecified)

df[, "Z"] <- rnorm(n = rows)

# generate confounder C (correctly specified)

df[, "C"] <- df[, "Z"]^2

# generate dichotomous exposure X

lpx <- ix + cx * df[, "Z"] + cx * df[, "C"]

prx <- 1/(1 + exp(-lpx))

df[, "X"] <- rbinom(n = rows, size = 1, prob = prx)

# generate continuous outcome Y

df[, "Y"] <- iy + xy * df[, "X"] + cy * df[, "Z"] + cy * df[, "C"] + rnorm(n =

1)

# return data frame

return(df)

rm(lpx, prx)

}

# define simulation parameters

seed <- 20220718

reps <- 1000

n <- c(200, 500, 1000)

ix <- 0

iy <- 0

confounder_effects <- c(-0.59, -0.39, -0.14, 0.14, 0.39, 0.59)

xy <- 0.59

# generate data sets for all parameters defined above and save each set in

# folder '220119 Step 1 - Generated datasets'

for(j in n){

for(k in confounder_effects){

df <- generate_data(seed = seed,

reps = reps,

n = j,

ix = ix,

iy = iy,

cx = k,

xy = xy,

cy = k)

# save each file in folder 'Step 1 - Generated datasets'

save(df, file = paste0("Step 1 - Generated datasets\\",

"n = ", j, ", cx = ", k, ", cy = ", k, ".RData"))

}

}

*Step 2 – Generate Models*

# in scenario 1, both the confounder-exposure and the confounder-outcome effect

# are correctly specified

scenario1 <- function(df){

estimates <- as.data.frame(matrix(NA, nrow = 1, ncol = 4))

# 1. multivariable regression analysis

model_multivar <- glm(Y ~ X + Z + C, data = df)

estimates[1, 1] <- model_multivar$coefficients[2]

# 2. covariate adjustment using the ps

ps <- predict(glm(X ~ Z + C, family = "binomial", data = df), type = "response")

model_covadj <- glm(Y ~ X + ps, data = df)

estimates[1, 2] <- model_covadj$coefficients[2]

# stabilized IPW

ipw <- ifelse(df$X == 1, 1/ps, 1/(1-ps))

sipw <- ipw/sum(ipw)

model_sipw <- glm(Y ~ X, weights = sipw, data = df)

estimates[1, 3] <- model_sipw$coefficients[2]

# DR estimation

model_dr <- glm(Y ~ X + Z + C, weights = sipw, data = df)

estimates[1, 4] <- model_dr$coefficients[2]

return(estimates)

}

# in scenario 2, the confounder-exposure effect is correctly specified and the

# confounder-outcome effect is misspecified

scenario2 <- function(df){

estimates <- as.data.frame(matrix(NA, nrow = 1, ncol = 4))

# 1. multivariable regression analysis

model_multivar <- glm(Y ~ X + Z, data = df)

estimates[1, 1] <- model_multivar$coefficients[2]

# 2. covariate adjustment using the ps

ps <- predict(glm(X ~ Z + C, family = "binomial", data = df), type = "response")

model_covadj <- glm(Y ~ X + ps, data = df)

estimates[1, 2] <- model_covadj$coefficients[2]

# stabilized IPW

ipw <- ifelse(df$X == 1, 1/ps, 1/(1-ps))

sipw <- ipw/sum(ipw)

model_sipw <- glm(Y ~ X, weights = sipw, data = df)

estimates[1, 3] <- model_sipw$coefficients[2]

# DR estimation

model_dr <- glm(Y ~ X + Z, weights = sipw, data = df)

estimates[1, 4] <- model_dr$coefficients[2]

return(estimates)

}

# in scenario 3, the confounder-exposure effect is misspecified and the

# confounder-outcome effect is correctly specified

scenario3 <- function(df){

estimates <- as.data.frame(matrix(NA, nrow = 1, ncol = 4))

# 1. multivariable regression analysis

model_multivar <- glm(Y ~ X + Z + C, data = df)

estimates[1, 1] <- model_multivar$coefficients[2]

# 2. covariate adjustment using the ps

ps <- predict(glm(X ~ Z, family = "binomial", data = df), type = "response")

model_covadj <- glm(Y ~ X + ps, data = df)

estimates[1, 2] <- model_covadj$coefficients[2]

# stabilized IPW

ipw <- ifelse(df$X == 1, 1/ps, 1/(1-ps))

sipw <- ipw/sum(ipw)

model_sipw <- glm(Y ~ X, weights = sipw, data = df)

estimates[1, 3] <- model_sipw$coefficients[2]

# DR estimation

model_dr <- glm(Y ~ X + Z + C, weights = sipw, data = df)

estimates[1, 4] <- model_dr$coefficients[2]

return(estimates)

}

# in scenario 4, both the confounder-exposure and the confounder-outcome effect

# are misspecified

scenario4 <- function(df){

estimates <- as.data.frame(matrix(NA, nrow = 1, ncol = 4))

# 1. multivariable regression analysis

model_multivar <- glm(Y ~ X + Z, data = df)

estimates[1, 1] <- model_multivar$coefficients[2]

# 2. covariate adjustment using the ps

ps <- predict(glm(X ~ Z, family = "binomial", data = df), type = "response")

model_covadj <- glm(Y ~ X + ps, data = df)

estimates[1, 2] <- model_covadj$coefficients[2]

# stabilized IPW

ipw <- ifelse(df$X == 1, 1/ps, 1/(1-ps))

sipw <- ipw/sum(ipw)

model_sipw <- glm(Y ~ X, weights = sipw, data = df)

estimates[1, 3] <- model_sipw$coefficients[2]

# DR estimation

model_dr <- glm(Y ~ X + Z, weights = sipw, data = df)

estimates[1, 4] <- model_dr$coefficients[2]

return(estimates)

}

# function generate_models returns for each repetition the simulation details

# and the estimated treatment effects

generate_models <- function(df){

# create data frame to store effect estimates in

effects <- data.frame(matrix(NA, nrow = max(df$repnr) * 4, ncol = 10))

colnames(effects) <- c("scenario",

"repnr",

"n",

"cx",

"xy",

"cy",

"coef_multivar",

"coef_covadj",

"coef_sipw",

"coef_dr")

# store simulation characteristics

effects$scenario <- rep(seq(c(1:4)), max(df$repnr))

effects$repnr <- rep(unique(df$repnr), each = 4)

effects$n <- unique(df$n)

effects$cx <- unique(df$cx)

effects$xy <- unique(df$xy)

effects$cy <- unique(df$cy)

# for loop to iterate through each repetition

repnr <- unique(effects$repnr)

for(i in repnr){

temp <- df[df$repnr == i, ]

# estimate exposure effects under each scenario

effects[effects$repnr == i & effects$scenario == 1, c(7:10)] <-

scenario1(temp)

effects[effects$repnr == i & effects$scenario == 2, c(7:10)] <-

scenario2(temp)

effects[effects$repnr == i & effects$scenario == 3, c(7:10)] <-

scenario3(temp)

effects[effects$repnr == i & effects$scenario == 4, c(7:10)] <-

scenario4(temp)

}

# return data frame with all simulation details and exposure effect estimates

return(effects)

}

# save path

path <- "Step 1 - Generated datasets\\"

# save all file names in files

files <- list.files(path = path,

pattern = "*.RData")

# START FOR LOOP - loop through each file in the folder

for(i in files){

# load the data into the environment

load(paste0(path, i))

# run function

effects <- generate_models(df)

# save each file in folder 'Step 2 - Generated models'

save(effects, file = Step 2 - Generated models\\", i))

}

*Step 3 – Model performance*

performance_measures <- function(data){

# for each scenario, all estimates will be stored in a matrix

performance <- matrix(NA, nrow = 4, ncol = 3)

colnames(performance) <- c("mean(b)",

"AB",

"RB")

rownames(performance) <- c("Multivariable regression analysis",

"Covariate adjustment using the PS",

"Standardized IPW",

"DR estimation")

# functions to calculate the performance measures

# 1. absolute bias

AB <- function(data, variable){

return(mean(variable - data$xy))

}

# 2. relative bias

RB <- function(data, variable){

return(mean((variable - data$xy)/data$xy))

}

# run for loop

for(i in unique(data$scenario)){

df <- data[data$scenario == i, ]

# mean exposure effect

performance[1, "mean(b)"] <- mean(df$coef_multivar)

performance[2, "mean(b)"] <- mean(df$coef_covadj)

performance[3, "mean(b)"] <- mean(df$coef_sipw)

performance[4, "mean(b)"] <- mean(df$coef_dr)

# absolute bias

performance[1, "AB"] <- AB(df, df$coef_multivar)

performance[2, "AB"] <- AB(df, df$coef_covadj)

performance[3, "AB"] <- AB(df, df$coef_sipw)

performance[4, "AB"] <- AB(df, df$coef_dr)

# relative bias

performance[1, "RB"] <- RB(df, df$coef_multivar)

performance[2, "RB"] <- RB(df, df$coef_covadj)

performance[3, "RB"] <- RB(df, df$coef_sipw)

performance[4, "RB"] <- RB(df, df$coef_dr)

# round to 4 digits

performance <- round(performance, 4)

# return scenario number and performance measures matrix

print(paste0("scenario number ", i))

print(performance)

}

}

# save path

path <- "Step 2 - Generated models\\"

# save all file names in files

files <- list.files(path = path,

pattern = "*.RData")

# START FOR LOOP - loop through each file in the folder

for(i in files){

# load the data into the environment

load(paste0(path, i))

# print scenario and all performance measures

print(i)

performance_measures(effects)

}
