# Supplementary material for: Misspecification of confounder-exposure and confounder-outcome associations leads to bias in effect estimates
Source: BMC Med Res Methodol. 2023 Jan 12;23:11. doi: 10.1186/s12874-022-01817-0 (PMC9835340; doi:10.1186/s12874-022-01817-0)
Supplement: Supplementary file 2 — Additional file 2. [file 12874_2022_1817_MOESM2_ESM.docx]

**Additional file B Model performance for sample size 200**

**Table B1** Model performance across all simulated scenarios, n = 200

|  | **Parameter values for the confounder-exposure and confounder-outcome associations** | | | | | | | | |
| --- | --- | --- | --- | --- | --- | --- | --- | --- | --- |
|  | 0.14 | | | 0.39 | | | 0.59 | | |
|  | $\hat{\beta}$ | AB | RB | $\hat{\beta}$ | AB | RB | $\hat{\beta}$ | AB | RB |
| **Scenario 1: correct specification of cx-association & correct specification of cy-association** | | | | | | | | | |
| Multivariable regression analysis | 0.5900 | 0.0000 | 0.0000 | 0.5900 | 0.0000 | 0.0000 | 0.5900 | 0.0000 | 0.0000 |
| Covariate adjustment using the PS | 0.5902 | 0.0002 | 0.0004 | 0.5917 | 0.0017 | 0.0029 | 0.5917 | 0.0017 | 0.0028 |
| Stabilized IPW | 0.5921 | 0.0021 | 0.0036 | 0.6182 | 0.0282 | 0.0478 | 0.6715 | 0.0815 | 0.1382 |
| DR estimation | 0.5900 | 0.0000 | 0.0000 | 0.5900 | 0.0000 | 0.0000 | 0.5900 | 0.0000 | 0.0000 |
| **Scenario 2: correct specification of cx-association & misspecification of cy-association** | | | | | | | | | |
| Multivariable regression analysis | 0.6256 | 0.0356 | 0.0603 | 0.8031 | 0.2132 | 0.3611 | 0.9813 | 0.3913 | 0.6632 |
| Covariate adjustment using the PS | 0.5902 | 0.0002 | 0.0004 | 0.5917 | 0.0017 | 0.0029 | 0.5917 | 0.0017 | 0.0028 |
| Stabilized IPW | 0.5921 | 0.0021 | 0.0036 | 0.6182 | 0.0282 | 0.0478 | 0.6715 | 0.0815 | 0.1382 |
| DR estimation | 0.5920 | 0.0020 | 0.0034 | 0.6201 | 0.0301 | 0.0510 | 0.6710 | 0.0810 | 0.1373 |
| **Scenario 3: misspecification of cx-association & correct specification of cy-association** | | | | | | | | | |
| Multivariable regression analysis | 0.5900 | 0.0000 | 0.0000 | 0.5900 | 0.0000 | 0.0000 | 0.5900 | 0.0000 | 0.0000 |
| Covariate adjustment using the PS | 0.6260 | 0.0360 | 0.0611 | 0.8127 | 0.2227 | 0.3774 | 1.0107 | 0.4207 | 0.7131 |
| Stabilized IPW | 0.6274 | 0.0374 | 0.0633 | 0.8323 | 0.2423 | 0.4107 | 1.0639 | 0.4739 | 0.8032 |
| DR estimation | 0.5900 | 0.0000 | 0.0000 | 0.5900 | 0.0000 | 0.0000 | 0.5900 | 0.0000 | 0.0000 |
| **Scenario 4: misspecification of cx-association & misspecification of cy-association** | | | | | | | | | |
| Multivariable regression analysis | 0.6256 | 0.0356 | 0.0603 | 0.8031 | 0.2131 | 0.3611 | 0.9813 | 0.3913 | 0.6632 |
| Covariate adjustment using the PS | 0.6260 | 0.0360 | 0.0611 | 0.8127 | 0.2227 | 0.3774 | 1.0107 | 0.4207 | 0.7131 |
| Stabilized IPW | 0.6274 | 0.0374 | 0.0633 | 0.8323 | 0.2423 | 0.4107 | 1.0639 | 0.4739 | 0.8032 |
| DR estimation | 0.6271 | 0.0371 | 0.0629 | 0.8250 | 0.2350 | 0.3983 | 1.0424 | 0.4524 | 0.7668 |

Abbreviations: n: sample size; cx-association: confounder-exposure association; cy-association: confounder-outcome association; $\hat{\beta}$: mean estimated exposure effect; AB: absolute bias;

RB: relative bias; PS: propensity score; IPW: inverse probability weighting; DR: double robust

**Table B2** Model performance across all simulated scenarios, n = 200

|  | **Parameter values for the confounder-exposure and confounder-outcome associations** | | | | | | | | |
| --- | --- | --- | --- | --- | --- | --- | --- | --- | --- |
|  | -0.14 | | | -0.39 | | | -0.59 | | |
|  | $\hat{\beta}$ | AB | RB | $\hat{\beta}$ | AB | RB | $\hat{\beta}$ | AB | RB |
| **Scenario 1: correct specification of cx-association & correct specification of cy-association** | | | | | | | | | |
| Multivariable regression analysis | 0.5900 | 0.0000 | 0.0000 | 0.5900 | 0.0000 | 0.0000 | 0.5900 | 0.0000 | 0.0000 |
| Covariate adjustment using the PS | 0.5902 | 0.0002 | 0.0003 | 0.5919 | 0.0019 | 0.0032 | 0.5930 | 0.0030 | 0.0051 |
| Stabilized IPW | 0.5913 | 0.0013 | 0.0022 | 0.6221 | 0.0321 | 0.0545 | 0.6671 | 0.0771 | 0.1307 |
| DR estimation | 0.5900 | 0.0000 | 0.0000 | 0.5900 | 0.0000 | 0.0000 | 0.5900 | 0.0000 | 0.0000 |
| **Scenario 2: correct specification of cx-association & misspecification of cy-association** | | | | | | | | | |
| Multivariable regression analysis | 0.6265 | 0.0365 | 0.0619 | 0.7999 | 0.2099 | 0.3558 | 0.9754 | 0.3854 | 0.6533 |
| Covariate adjustment using the PS | 0.5902 | 0.0002 | 0.0003 | 0.5919 | 0.0019 | 0.0032 | 0.5930 | 0.0030 | 0.0051 |
| Stabilized IPW | 0.5913 | 0.0013 | 0.0022 | 0.6221 | 0.0321 | 0.0545 | 0.6671 | 0.0771 | 0.1307 |
| DR estimation | 0.5917 | 0.0017 | 0.0029 | 0.6216 | 0.0316 | 0.0536 | 0.6717 | 0.0817 | 0.1384 |
| **Scenario 3: misspecification of cx-association & correct specification of cy-association** | | | | | | | | | |
| Multivariable regression analysis | 0.5900 | 0.0000 | 0.0000 | 0.5900 | 0.0000 | 0.0000 | 0.5900 | 0.0000 | 0.0000 |
| Covariate adjustment using the PS | 0.6270 | 0.0370 | 0.0627 | 0.8105 | 0.2205 | 0.3737 | 1.0042 | 0.4142 | 0.7020 |
| Stabilized IPW | 0.6283 | 0.0383 | 0.0648 | 0.8320 | 0.2420 | 0.4102 | 1.0561 | 0.4661 | 0.7900 |
| DR estimation | 0.5900 | 0.0000 | 0.0000 | 0.5900 | 0.0000 | 0.0000 | 0.5900 | 0.0000 | 0.0000 |
| **Scenario 4: misspecification of cx-association & misspecification of cy-association** | | | | | | | | | |
| Multivariable regression analysis | 0.6265 | 0.0365 | 0.0619 | 0.7999 | 0.2099 | 0.3558 | 0.9754 | 0.3854 | 0.6533 |
| Covariate adjustment using the PS | 0.6270 | 0.0370 | 0.0627 | 0.8105 | 0.2205 | 0.3737 | 1.0042 | 0.4142 | 0.7020 |
| Stabilized IPW | 0.6283 | 0.0383 | 0.0648 | 0.8320 | 0.2420 | 0.4102 | 1.0561 | 0.4661 | 0.7900 |
| DR estimation | 0.6280 | 0.0380 | 0.0644 | 0.8239 | 0.2339 | 0.3965 | 1.0350 | 0.4450 | 0.7543 |

Abbreviations: n: sample size; cx-association: confounder-exposure association; cy-association: confounder-outcome association; $\hat{\beta}$: mean estimated exposure effect;

AB: absolute bias; RB: relative bias; PS: propensity score; IPW: inverse probability weighting; DR: double robust
