# Supplementary material for: Misspecification of confounder-exposure and confounder-outcome associations leads to bias in effect estimates
Source: BMC Med Res Methodol. 2023 Jan 12;23:11. doi: 10.1186/s12874-022-01817-0 (PMC9835340; doi:10.1186/s12874-022-01817-0)
Supplement: Supplementary file 3 — Additional file 3. [file 12874_2022_1817_MOESM3_ESM.docx]

**Additional file C Model performance for sample size 1,000**

**Table C1** Model performance across all simulated scenarios, n = 1,000

|  | **Parameter values for the confounder-exposure and confounder-outcome associations** | | | | | | | | |
| --- | --- | --- | --- | --- | --- | --- | --- | --- | --- |
|  | 0.14 | | | 0.39 | | | 0.59 | | |
|  | $\hat{\beta}$ | AB | RB | $\hat{\beta}$ | AB | RB | $\hat{\beta}$ | AB | RB |
| **Scenario 1: correct specification of cx-association & correct specification of cy-association** | | | | | | | | | |
| Multivariable regression analysis | 0.5900 | 0.0000 | 0.0000 | 0.5900 | 0.0000 | 0.0000 | 0.5900 | 0.0000 | 0.0000 |
| Covariate adjustment using the PS | 0.5900 | 0.0000 | 0.0000 | 0.5903 | 0.0003 | 0.0006 | 0.5908 | 0.0008 | 0.0013 |
| Stabilized IPW | 0.5902 | 0.0002 | 0.0003 | 0.5971 | 0.0071 | 0.0120 | 0.6212 | 0.0312 | 0.0528 |
| DR estimation | 0.5900 | 0.0000 | 0.0000 | 0.5900 | 0.0000 | 0.0000 | 0.5900 | 0.0000 | 0.0000 |
| **Scenario 2: correct specification of cx-association & misspecification of cy-association** | | | | | | | | | |
| Multivariable regression analysis | 0.6263 | 0.0363 | 0.0615 | 0.8027 | 0.2127 | 0.3606 | 0.9863 | 0.3963 | 0.6718 |
| Covariate adjustment using the PS | 0.5900 | 0.0000 | 0.0000 | 0.5903 | 0.0003 | 0.0006 | 0.5908 | 0.0008 | 0.0013 |
| Stabilized IPW | 0.5902 | 0.0002 | 0.0003 | 0.5971 | 0.0071 | 0.0120 | 0.6212 | 0.0312 | 0.0528 |
| DR estimation | 0.5903 | 0.0003 | 0.0004 | 0.6002 | 0.0102 | 0.0173 | 0.6277 | 0.0377 | 0.0639 |
| **Scenario 3: misspecification of cx-association & correct specification of cy-association** | | | | | | | | | |
| Multivariable regression analysis | 0.5900 | 0.0000 | 0.0000 | 0.5900 | 0.0000 | 0.0000 | 0.5900 | 0.0000 | 0.0000 |
| Covariate adjustment using the PS | 0.6267 | 0.0367 | 0.0621 | 0.8124 | 0.2224 | 0.3770 | 1.0145 | 0.4245 | 0.7195 |
| Stabilized IPW | 0.6275 | 0.0375 | 0.0636 | 0.8316 | 0.2416 | 0.4095 | 1.0640 | 0.4740 | 0.8033 |
| DR estimation | 0.5900 | 0.0000 | 0.0000 | 0.5900 | 0.0000 | 0.0000 | 0.5900 | 0.0000 | 0.0000 |
| **Scenario 4: misspecification of cx-association & misspecification of cy-association** | | | | | | | | | |
| Multivariable regression analysis | 0.6263 | 0.0363 | 0.0615 | 0.8027 | 0.2127 | 0.3606 | 0.9863 | 0.3963 | 0.6718 |
| Covariate adjustment using the PS | 0.6267 | 0.0367 | 0.0621 | 0.8124 | 0.2224 | 0.3770 | 1.0145 | 0.4245 | 0.7195 |
| Stabilized IPW | 0.6275 | 0.0375 | 0.0636 | 0.8316 | 0.2416 | 0.4095 | 1.0640 | 0.4740 | 0.8033 |
| DR estimation | 0.6272 | 0.0372 | 0.0631 | 0.8237 | 0.2337 | 0.3960 | 1.0423 | 0.4523 | 0.7667 |

Abbreviations: n: sample size; cx-association: confounder-exposure association; cy-association: confounder-outcome association; $\hat{\beta}$: mean estimated exposure effect; AB: absolute bias;

RB: relative bias; PS: propensity score; IPW: inverse probability weighting; DR: double robust

**Table C2** Model performance across all simulated scenarios, n = 1000

|  | **Parameter values for the confounder-exposure and confounder-outcome associations** | | | | | | | | |
| --- | --- | --- | --- | --- | --- | --- | --- | --- | --- |
|  | -0.14 | | | -0.39 | | | -0.59 | | |
|  | $\hat{\beta}$ | AB | RB | $\hat{\beta}$ | AB | RB | $\hat{\beta}$ | AB | RB |
| **Scenario 1: correct specification of cx-association & correct specification of cy-association** | | | | | | | | | |
| Multivariable regression analysis | 0.5900 | 0.0000 | 0.0000 | 0.5900 | 0.0000 | 0.0000 | 0.5900 | 0.0000 | 0.0000 |
| Covariate adjustment using the PS | 0.5900 | 0.0000 | 0.0001 | 0.5904 | 0.0004 | 0.0006 | 0.5905 | 0.0005 | 0.0009 |
| Stabilized IPW | 0.5903 | 0.0003 | 0.0005 | 0.5980 | 0.0080 | 0.0136 | 0.6188 | 0.0288 | 0.0489 |
| DR estimation | 0.5900 | 0.0000 | 0.0000 | 0.5900 | 0.0000 | 0.0000 | 0.5900 | 0.0000 | 0.0000 |
| **Scenario 2: correct specification of cx-association & misspecification of cy-association** | | | | | | | | | |
| Multivariable regression analysis | 0.6260 | 0.0360 | 0.0611 | 0.8030 | 0.2130 | 0.3610 | 0.9859 | 0.3959 | 0.6711 |
| Covariate adjustment using the PS | 0.5900 | 0.0000 | 0.0001 | 0.5904 | 0.0004 | 0.0006 | 0.5905 | 0.0005 | 0.0009 |
| Stabilized IPW | 0.5903 | 0.0003 | 0.0005 | 0.5980 | 0.0080 | 0.0136 | 0.6188 | 0.0288 | 0.0489 |
| DR estimation | 0.5904 | 0.0004 | 0.0006 | 0.6013 | 0.0113 | 0.0192 | 0.6286 | 0.0386 | 0.0654 |
| **Scenario 3: misspecification of cx-association & correct specification of cy-association** | | | | | | | | | |
| Multivariable regression analysis | 0.5900 | 0.0000 | 0.0000 | 0.5900 | 0.0000 | 0.0000 | 0.5900 | 0.0000 | 0.0000 |
| Covariate adjustment using the PS | 0.6264 | 0.0364 | 0.0617 | 0.8126 | 0.2226 | 0.3773 | 1.0141 | 0.4241 | 0.7189 |
| Stabilized IPW | 0.6273 | 0.0373 | 0.0632 | 0.8315 | 0.2415 | 0.4094 | 1.0635 | 0.4735 | 0.8025 |
| DR estimation | 0.5900 | 0.0000 | 0.0000 | 0.5900 | 0.0000 | 0.0000 | 0.5900 | 0.0000 | 0.0000 |
| **Scenario 4: misspecification of cx-association & misspecification of cy-association** | | | | | | | | | |
| Multivariable regression analysis | 0.6260 | 0.0360 | 0.0611 | 0.8030 | 0.2130 | 0.3610 | 0.9859 | 0.3959 | 0.6711 |
| Covariate adjustment using the PS | 0.6264 | 0.0364 | 0.0617 | 0.8126 | 0.2226 | 0.3773 | 1.0141 | 0.4241 | 0.7189 |
| Stabilized IPW | 0.6273 | 0.0373 | 0.0632 | 0.8315 | 0.2415 | 0.4094 | 1.0635 | 0.4735 | 0.8025 |
| DR estimation | 0.6270 | 0.0370 | 0.0627 | 0.8237 | 0.2337 | 0.3962 | 1.0420 | 0.4520 | 0.7661 |

Abbreviations: n: sample size; cx-association: confounder-exposure association; cy-association: confounder-outcome association; $\hat{\beta}$: mean estimated exposure effect;

AB: absolute bias; RB: relative bias; PS: propensity score; IPW: inverse probability weighting; DR: double robust
