# Supplementary material for: Misspecification of confounder-exposure and confounder-outcome associations leads to bias in effect estimates
Source: BMC Med Res Methodol. 2023 Jan 12;23:11. doi: 10.1186/s12874-022-01817-0 (PMC9835340; doi:10.1186/s12874-022-01817-0)
Supplement: Supplementary file 4 — Additional file 4. [file 12874_2022_1817_MOESM4_ESM.docx]

**Additional file D Model performance for sample size 500**

**Table D1** Model performance across all simulated scenarios, n = 500

|  | **Parameter values for the confounder-exposure and confounder-outcome associations** | | | | | | | | |
| --- | --- | --- | --- | --- | --- | --- | --- | --- | --- |
|  | -0.14 | | | -0.39 | | | -0.59 | | |
|  | $\hat{\beta}$ | AB | RB | $\hat{\beta}$ | AB | RB | $\hat{\beta}$ | AB | RB |
| **Scenario 1: correct specification of cx-association & correct specification of cy-association** | | | | | | | | | |
| Multivariable regression analysis | 0.5900 | 0.0000 | 0.0000 | 0.5900 | 0.0000 | 0.0000 | 0.5900 | 0.0000 | 0.0000 |
| Covariate adjustment using the PS | 0.5901 | 0.0001 | 0.0001 | 0.5909 | 0.0009 | 0.0015 | 0.5914 | 0.0014 | 0.0023 |
| Stabilized IPW | 0.5900 | 0.0000 | 0.0000 | 0.6092 | 0.0192 | 0.0326 | 0.6444 | 0.0544 | 0.0922 |
| DR estimation | 0.5900 | 0.0000 | 0.0000 | 0.5900 | 0.0000 | 0.0000 | 0.5900 | 0.0000 | 0.0000 |
| **Scenario 2: correct specification of cx-association & misspecification of cy-association** | | | | | | | | | |
| Multivariable regression analysis | 0.6259 | 0.0359 | 0.0608 | 0.8031 | 0.2131 | 0.3612 | 0.9882 | 0.3982 | 0.6750 |
| Covariate adjustment using the PS | 0.5901 | 0.0001 | 0.0001 | 0.5909 | 0.0009 | 0.0015 | 0.5914 | 0.0014 | 0.0023 |
| Stabilized IPW | 0.5900 | 0.0000 | 0.0000 | 0.6092 | 0.0192 | 0.0326 | 0.6444 | 0.0544 | 0.0922 |
| DR estimation | 0.5904 | 0.0004 | 0.0007 | 0.6094 | 0.0194 | 0.0330 | 0.6460 | 0.0560 | 0.0949 |
| **Scenario 3: misspecification of cx-association & correct specification of cy-association** | | | | | | | | | |
| Multivariable regression analysis | 0.5900 | 0.0000 | 0.0000 | 0.5900 | 0.0000 | 0.0000 | 0.5900 | 0.0000 | 0.0000 |
| Covariate adjustment using the PS | 0.6263 | 0.0363 | 0.0615 | 0.8130 | 0.2230 | 0.3780 | 1.0168 | 0.4268 | 0.7234 |
| Stabilized IPW | 0.6272 | 0.0372 | 0.0631 | 0.8329 | 0.2429 | 0.4117 | 1.0671 | 0.4771 | 0.8086 |
| DR estimation | 0.5900 | 0.0000 | 0.0000 | 0.5900 | 0.0000 | 0.0000 | 0.5900 | 0.0000 | 0.0000 |
| **Scenario 4: misspecification of cx-association & misspecification of cy-association** | | | | | | | | | |
| Multivariable regression analysis | 0.6259 | 0.0359 | 0.0608 | 0.8031 | 0.2131 | 0.3612 | 0.9882 | 0.3982 | 0.6750 |
| Covariate adjustment using the PS | 0.6263 | 0.0363 | 0.0615 | 0.8130 | 0.2230 | 0.3780 | 1.0168 | 0.4268 | 0.7234 |
| Stabilized IPW | 0.6272 | 0.0372 | 0.0631 | 0.8329 | 0.2429 | 0.4117 | 1.0671 | 0.4771 | 0.8086 |
| DR estimation | 0.6269 | 0.0369 | 0.0626 | 0.8250 | 0.2350 | 0.3983 | 1.0451 | 0.4551 | 0.7714 |

Abbreviations: n: sample size; cx-association: confounder-exposure association; cy-association: confounder-outcome association; $\hat{\beta}$: mean estimated exposure effect;

AB: absolute bias; RB: relative bias; PS: propensity score; IPW: inverse probability weighting; DR: double robust
