# Supplementary material for: Misspecification of confounder-exposure and confounder-outcome associations leads to bias in effect estimates
Source: BMC Med Res Methodol. 2023 Jan 12;23:11. doi: 10.1186/s12874-022-01817-0 (PMC9835340; doi:10.1186/s12874-022-01817-0)
Supplement: Supplementary file 5 — Additional file 5. [file 12874_2022_1817_MOESM5_ESM.docx]

**Additional file E Sign change as a results of misspecification of the confounder-exposure and confounder-outcome associations**

In some scenarios, incorrect modelling of non-linear confounder-exposure and confounder-outcome associations may lead to a change of the direction of the exposure effect. In this illustration, we consider an exposure effect of -0.14 (a small negative effect), and confounder-exposure and confounder-outcome associations of 0.59 (a large positive effect). The sample size and the number of repetitions are both 1,000.

Table E1 shows that, if the confounder-exposure association is misspecified (scenario 2), multivariable regression analysis results in a positive exposure effect estimate. If the confounder-outcome association is misspecified, a sign change occurs for covariate adjustment using the PS and stabilized IPW. If both associations are misspecified, all methods estimate a positive exposure effect.

**Table E1** Model performance across simulated scenarios for sample size 1000 and exposure effect -0.14

|  | Parameter value for the confounder-exposure and confounder-outcome associations: 0.59 | | |
| --- | --- | --- | --- |
|  | $\hat{\beta}$ | AB | RB |
| **Scenario 1: correct specification of cx-association & correct specification of cy-association** | | | |
| Multivariable regression analysis | -0.1400 | 0.0000 | 0.0000 |
| Covariate adjustment using the PS | -0.1402 | -0.0002 | 0.0016 |
| Stabilized IPW | -0.1271 | 0.0129 | -0.0925 |
| DR estimation | -0.1400 | 0.0000 | 0.0000 |
| **Scenario 2: correct specification of cx-association & misspecification of cy-association** | | | |
| Multivariable regression analysis | 0.2556 | 0.3956 | -2.8261 |
| Covariate adjustment using the PS | -0.1402 | -0.0002 | 0.0016 |
| Stabilized IPW | -0.1471 | 0.0129 | -0.0925 |
| DR estimation | -0.1087 | 0.0313 | -0.2236 |
| **Scenario 3: misspecification of cx-association & correct specification of cy-association** | | | |
| Multivariable regression analysis | -0.1400 | 0.0000 | 0.0000 |
| Covariate adjustment using the PS | 0.2844 | 0.4244 | -3.0313 |
| Stabilized IPW | 0.3339 | 0.4739 | -3.3849 |
| DR estimation | -0.1400 | 0.0000 | 0.0000 |
| **Scenario 4: misspecification of cx-association & misspecification of cy-association** | | | |
| Multivariable regression analysis | 0.2556 | 0.3956 | -2.8261 |
| Covariate adjustment using the PS | 0.2844 | 0.4244 | -3.0313 |
| Stabilized IPW | 0.3339 | 0.4739 | -3.3849 |
| DR estimation | 0.3120 | 0.4520 | -3.2288 |
